# Bioinformatic challenges for proteomic biomarkers of cancer

**DOI:** 10.1186/1471-2105-12-S7-A17

**Published:** 2011-08-05

**Authors:** David L Tabb, Daniel C Liebler

**Affiliations:** 1 Biomedical Informatics, Vanderbilt University, Nashville, TN, 37232-8575, USA; 2 Biochemistry, Vanderbilt University, Nashville, TN, 37232-6350, USA

## Background

Proteomic biomarkers are sets of proteins that may be detected in biofluids for disease detection, prognosis, and treatment selection. Vanderbilt University is taking part in two National Cancer Institute initiatives to establish a basis for clinical biomarkers. The first, Clinical Proteomic Technology Assessment for Cancer (CPTAC), characterizes proteomic technologies for discovery and validation of biomarkers. The second, the Early Detection Research Network (EDRN), attempts to develop new sets of biomarkers and evaluate their effectiveness in clinically relevant samples. The quest for biomarkers is fraught with challenges for both “bench” experiments and bioinformatics. Here, we examine sources of discrepancy at several levels. The search engines that identify peptides from LC-MS/MS experiments differ significantly in the set of spectra that they identify from experiments. Parsimonious protein assembly may prune out proteins in one experiment and retain them in another. Protein differentiation tools may yield divergent results, even when starting from the same data sets. Verifying biomarkers through targeted proteomics has only recently been supported by tools that can work across instruments from different vendors. Translating cancer biology knowledge into clinically relevant tests for this disease will require care at all these levels.

